# Synergistic Nisin-Polymyxin Combinations for the Control of *Pseudomonas* Biofilm Formation

**DOI:** 10.3389/fmicb.2016.01713

**Published:** 2016-10-26

**Authors:** Des Field, Nynke Seisling, Paul D. Cotter, R. P. Ross, Colin Hill

**Affiliations:** ^1^School of Microbiology, University College CorkCork, Ireland; ^2^Teagasc Food Research CentreCork, Ireland; ^3^APC Microbiome Institute, University College CorkCork, Ireland

**Keywords:** biofilm, colistin, polymyxin, nisin, lantibiotic, *P. aeruginosa*, bacteriocin, antibiotics

## Abstract

The emergence and dissemination of multi-drug resistant pathogens is a global concern. Moreover, even greater levels of resistance are conferred on bacteria when in the form of biofilms (i.e., complex, sessile communities of bacteria embedded in an organic polymer matrix). For decades, antimicrobial peptides have been hailed as a potential solution to the paucity of novel antibiotics, either as natural inhibitors that can be used alone or in formulations with synergistically acting antibiotics. Here, we evaluate the potential of the antimicrobial peptide nisin to increase the efficacy of the antibiotics polymyxin and colistin, with a particular focus on their application to prevent biofilm formation of *Pseudomonas aeruginosa*. The results reveal that the concentrations of polymyxins that are required to effectively inhibit biofilm formation can be dramatically reduced when combined with nisin, thereby enhancing efficacy, and ultimately, restoring sensitivity. Such combination therapy may yield added benefits by virtue of reducing polymyxin toxicity through the administration of significantly lower levels of polymyxin antibiotics.

## Introduction

The increasing spread of antibiotic resistance in Gram-negative bacteria, particularly in *Pseudomonas aeruginosa, Acinetobacter baumannii* and *Klebsiella pneumoniae*, represents a major global medical challenge (Bergen et al., 2012). Mortality, morbidity, and health care costs are substantially increased as a result of infections caused by these pathogens (Boucher et al., 2009). The situation is exacerbated by the lack of progress with respect to the clinical development of new antibiotics for Gram-negative bacteria over the last few decades (Carlet et al., 2012). These factors have led to a revival in the use of polymyxins to treat recalcitrant infections that are resistant to most or all other currently available antibiotics. In clinical settings, colistin (i.e., polymyxin E) and polymyxin B were initially used to treat numerous infections caused by Gram-negative bacteria, including sepsis, wound infections, urinary tract infection, pneumonia, and catheter-based infections (Landman et al., 2008). Polymyxins exert their antimicrobial action via direct interaction with the lipid A component of the lipopolysaccharide (LPS), resulting in the increased permeability of the bacterial cell membrane (Velkov et al., 2010). Although introduced in the 1950s, colistin and polymyxin B were abandoned in the 1970s due to reports of serious toxic effects, mainly to the kidney and nervous system (Velkov et al., 2013). However, the rapid increase in resistance to all other antibiotics necessitated their re-evaluation and in the 1980s colistin was reintroduced to control infection or colonization by *P. aeruginosa* in patients with cystic fibrosis (CF) (Nation and Li, 2009). Despite their relatively recent reintegration in clinical practice, microbial resistance is already an issue of significance, with reports of the existence of plasmid-borne polymyxin resistance determinants (Liu et al., 2016) potentiating the rapid spread of resistance to these last-line antibiotics. Furthermore, antibiotic therapy by these, and other antibiotics, is hindered by the innate antibiotic resistance of bacteria present in biofilms (complex, sessile communities of bacteria embedded in an organic polymer matrix), making novel anti-biofilm strategies highly desirable (Marcinkiewicz et al., 2013). Approaches to overcome these issues, including drug discovery programs for the development of new polymyxin derivatives that are safer and more efficacious, have met with little success (Velkov et al., 2016). An alternative option is the use of polymyxins in combination with other antimicrobial agents including peptide inhibitors. Indeed, such strategies for peptide-antibiotic combinations to address issues related to prevent and eradicate bacterial biofilms formed by multidrug-resistant bacteria show great promise (Reffuveille et al., 2014; de la Fuente-Núñez and Hancock, 2015). In keeping with this line of enquiry, there has been a particular focus on assessing and enhancing the benefits of applying lantibiotics in clinical settings (Cotter et al., 2013; Field et al., 2015a). Lantibiotics are ribosomally synthesized peptides that are distinguished by the presence of unusual amino acids including lanthionine and/or methyllanthionine (Breukink and de Kruijff, 1999; Bierbaum and Sahl, 2009), and have become the focus of much biomedical and pharmaceutical research due to their high potency *in vitro*, numerous modes of action and capacity to destroy target cells rapidly (Cotter et al., 2005; Cavera et al., 2015). The most thoroughly investigated lantibiotic is nisin, a 34 amino acid polycyclic peptide that exhibits antibacterial activity against a wide range of clinical and food-borne pathogens that is widely used as a natural biopreservative (Delves-Broughton et al., 1996; Deegan et al., 2006). It has frequently been suggested that the efficacy of nisin could be further improved through combination with other antimicrobials or membrane-active substances (Cavera et al., 2015; Field et al., 2015b). Indeed, several studies have demonstrated synergistic relationships between conventional antibiotics and nisin. The majority of these studies have involved Gram-positive bacteria such as staphylococci, including methicillin-resistant forms (Piper et al., 2009; Dosler and Gerceker, 2011; Okuda et al., 2013), enterococci (Tong et al., 2014), including vancomycin-resistant enterococci (Brumfitt et al., 2002), and streptococci (Lebel et al., 2013). Nisin-antibiotic combinations have also been shown to be effective against Gram-positive bacterial biofilms (Okuda et al., 2013; Field et al., 2015c). Recent combinatorial nisin-antibiotic investigations have been directed against Gram-negative bacteria. For example, nisin displayed synergistic activity with the antibiotics penicillin, streptomycin, chloramphenicol and rifampicin against *P. fluorescens* (Naghmouchi et al., 2012), and with colistin against *Salmonella choleraesuis, P. aeruginosa, Yersinia enterocolitica*, and *Escherichia coli* (Naghmouchi et al., 2013). Similarly, nisin-ceftriaxone and nisin-cefotaxime were found to be highly synergistic when applied against clinical isolates of *S.* *enterica* serovar Typhimurium, as evident by checkerboard and time-kill assays (Rishi et al., 2014). While synergistic *in vitro* activities of antibiotics and antimicrobial cationic peptides in combination against biofilms of *P. aeruginosa* have been demonstrated (Dosler and Karaaslan, 2014), the effects of the prototypical lantibiotic nisin and antibiotic combinations on biofilm formation of Gram-negative bacteria has not been investigated. Here we assess the impact of combining nisin with a variety of clinical antibiotics and establish that nisin exhibits enhanced inhibitory activity in combination with either polymyxin B or colistin. Furthermore, we reveal that the combinations are more effective at inhibiting *P. aeruginosa* biofilm formation compared to when either antimicrobial is used alone. Importantly, the results provide data on effective synergistic concentrations that may allow for the effective clinical use of significantly lower levels of the nephrotoxic antibiotics colistin and polymyxin B

## Materials and Methods

### Bacterial Strains and Growth Conditions

*Lactococcus lactis* NZ9700 was grown in M17 broth supplemented with 0.5% glucose (GM17) or GM17 agar at 30°C. *E. coli, K. pneumoniae* and *Pseudomonas* strains were grown in Luria–Bertani (LB) broth [5 g L^-1^ yeast extract (Oxoid), 10 g L^-1^ tryptone (Oxoid) and 10 g L^-1^ NaCl (Merck)], incubated overnight at 37°C and shaken at 170 rpm.

### Minimum Inhibitory Concentration Assays

Minimum inhibitory concentration (MIC) determinations were carried out in triplicate in 96 well microtiter plates as described previously (Field et al., 2010, 2012). Briefly, target strains were grown overnight in the appropriate conditions and medium, subcultured into fresh broth and allowed to grow to an OD_600_ of ∼0.5, diluted to a final concentration of 10^5^ cfu ml^-1^ in a volume of 0.2 ml. Chloramphenicol, penicillin G, erythromycin, colistin, and polymyxin B (Sigma) were resuspended in LB media to a stock concentration of 128 or 256 μg/ml. The antibiotics were adjusted to 16, 32, 64, or 128 μg/ml starting concentration and twofold serial dilutions of each compound were made in 96 well plates for a total of 12 dilutions. Purified nisin was adjusted to a 100 μM (when using *E. coli, K. pneumoniae*, and *Pseudomonas putida* as a target) or 200 μM (*P. aeruginosa*) starting concentration and twofold serial dilutions of each peptide were carried out. The target strain was then added and after incubation for 16 h at 37°C and the MIC was read as the lowest peptide concentration causing inhibition of visible growth.

### Nisin Purification

Nisin was purified according to previously described protocols (Field et al., 2010; Healy et al., 2013). The purified nisin peptide was subjected to MALDI-ToF Mass Spectrometric analysis to confirm purity before use.

### Growth Curve Experiments

For growth experiments, overnight cultures were transferred (10^7^ cfu ml^-1^ in a volume of 1.0 ml.) into LB supplemented with the relevant concentration of nisin A and antibiotic/peptide combinations, and subsequently 0.2 ml was transferred to 96 well microtiter plates (Sarstedt). Cell growth was measured spectrophotometrically over 24 or 48-h periods by using a SpectraMax M3 spectrophotometer (Molecular Devices, Sunnyvale, CA, USA).

### Biofilm Formation

Static microtiter plate assays based on a previous study (Kelly et al., 2012), but with modifications to optimize the assay. Briefly, a 1:100 dilution was performed by adding 2 μl of log phase cells (10^7^ CFU ml^-1^ of each culture) to 198 μl of LB in wells of a sterile 96-well microtiter plate (Sarstedt, Leicester, UK), giving a starting inoculum of 10^5^ CFU ml^-1^; 200 μl of LB was added to a set of wells as a negative control. All wells were seeded in triplicate. Microtiter plates were then incubated at 37°C for 24 h to allow biofilm formation to occur and Washing (PBS) and staining of wells (0.05% crystal violet) was carried out as described previously (Field et al., 2015c).

### Inhibition of Biofilm Formation

Antibiotics (colistin or polymyxin) were added to the microtiter plate wells at 1/2×, 1/5×, 1/10×, and nisin peptide at 1/3× or 1/10× and combinations thereof the relevant MIC as previously determined. Log phase cells were added to give a starting inoculum of 10^5^ CFU ml^-1^; all wells were seeded in triplicate. The plate was incubated for 24 h, at 37°C and cell growth measured using a SpectraMax M3 spectrophotometer (Molecular Devices, Sunnyvale, CA, USA). The plates were removed and washing (PBS) and staining of wells (0.05% crystal violet) was carried out as described previously (Field et al., 2015c). Absorbance was measured at 595 nm using a microtiter plate reader (Molecular Devices Spectramax M3, Sunnyvale, CA, USA). Data obtained in triplicate were calculated and expressed as the mean ± standard deviations.

## Results

### Bacterial Susceptibility to Antimicrobial Compounds

Minimum inhibitory concentration with purified nisin A peptide, as well as a range of antibiotics including penicillin, erythromycin, chloramphenicol, colistin, and polymyxin B, were carried out to establish suitable concentrations for combinatorial studies with nisin against the Gram-negative targets *E. coli* K12 MG1655, *K. pneumoniae* NCIMB 13218, *P. putida* CA-3, and *P. aeruginosa* PA-01. Activity against the target strains required a relatively high concentration of nisin (50–200 μg/ml). These values were in agreement with data obtained by Naghmouchi et al. (2013) against a panel of Gram-negative strains and, yet again, highlights the relative resistance of Gram-negative bacteria to nisin compared to Gram-positive strains, with some examples of the latter having MICs in the nanomolar (nM) range. *E. coli, Klebsiella and Pseudomonas* strains were relatively resistant to erythromycin and penicillin but, with the exception of *K. pneumoniae* NCIMB 13218, were sensitive to chloramphenicol. MICs for colistin and polymyxin B against *E. coli* K12 MG1655 were in close agreement with previously established figures against strains of *E. coli* (Corvec et al., 2013). Similarly, colistin and polymyxin B exhibited almost identical activity (Gales et al., 2011) against *K. pneumoniae* NCIMB 13218 and the *Pseudomonas* strains and were within previously established ranges (MIC_90_, 2 μg/ml) (Gales et al., 2001).

### Growth Curve-Based Comparisons of the Activity of Nisin A and Antibiotic Combinations

**FIGURE 1:**
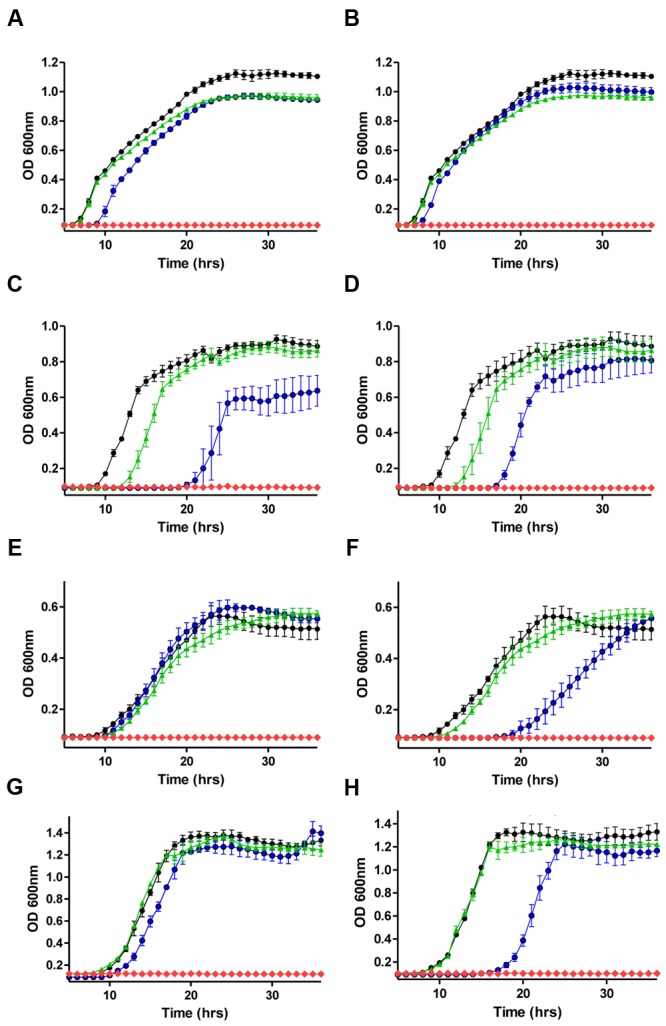
**Growth curve analysis of **(A)***E. coli* MG1655 in the presence of 1/5× minimum inhibitory concentration (MIC; 10 μg/ml) nisin A (green triangle), 1/8× MIC (0.05 μg/ml) colistin (blue circle), in combination (red diamond) and untreated control (black circle) **(B)** 1/5× MIC (10 μg/ml) nisin A (green triangle), 1/8× MIC (0.05 μg/ml) polymyxin B (blue circle) in combination (red diamond), **(C)***Klebsiella pneumoniae* NCIMB 13218 in the presence of 1/3× MIC (16.66 μg/ml) nisin A (green triangle), 1/2× MIC (0.75 μg/ml) colistin (blue circle), in combination (red diamond) and untreated control (black circle), **(D)** 1/3× MIC (16.66 μg/ml) nisin A (green triangle), 1/2× MIC (0.75 μg/ml) polymyxin (blue circle), in combination (red diamond) and untreated control (black circle), **(E)***Pseudomonas putida* CA-3 in the presence of 1/3× MIC (16.66 μg/ml) nisin A (green triangle), 1/4× MIC (0.1 μg/ml) colistin (blue circle), in combination (red diamond) and untreated control (black circle), **(F)** 1/3× MIC (16.66 μg/ml) nisin A (green triangle), 1/4× MIC (0.2 μg/ml) polymyxin B (blue circle), in combination (red diamond) and untreated control (black circle), and **(G)***P. aeruginosa* PA-01 in the presence of 1/4× MIC (50 μg/ml) nisin A (green triangle), 1/2× MIC (0.75 μg/ml) colistin (blue circle), in combination (red diamond) and untreated control (black circle), **(H)** 1/4× MIC (50 μg/ml) nisin A (green triangle), 1/2× MIC (0.75 μg/ml) polymyxin B (blue circle), in combination (red diamond) and untreated control (black circle)**.

Having established the MIC values for nisin A and a range of antibiotics against the representative Gram-negative strains, growth curves were performed in order to reveal the impact of sub-lethal concentrations of nisin A and antibiotics (alone and in combination) on bacterial growth. The final concentration of nisin or antibiotic used for each organism was a fraction of the previously determined MIC value (i.e., 1/2×, 1/3×, 1/4×, etc.) and combinations thereof. It was decided that penicillin and chloramphenicol should be included for combinatorial analysis given previous reports of synergism between these antibiotics and nisin A against strains of *Pseudomonas* (Naghmouchi et al., 2013). When nisin + penicillin, nisin + erythromycin or nisin + chloramphenicol combinations were employed against *E. coli* K12 MG1655, *K. pneumoniae* NCIMB 13218, *P. putida* CA-3, and *P. aeruginosa* PA-01, little to no synergistic effects were observed at the sub-inhibitory concentrations used (data not shown). However, pronounced inhibitory effects were observed when colistin or polymyxin B was combined with nisin, compared to the untreated control or when each of the antimicrobials was used alone (**Figure 1**). In the case of *E. coli* K12 MG1655, a combination of 1/8× MIC (0.05 μg/ml) colistin or polymyxin and 1/5× MIC (10 μg/ml) nisin A resulted in complete inhibition of growth (**Figures 1A,B**). Similarly, no growth of *K. pneumoniae* was observed when 1/2× MIC (0.75 μg/ml) of either colistin or polymyxin was used in combination with 1/3× MIC (16.66 μg/ml) nisin A (**Figures 1C,D**). Nisin at 1/3× MIC (16.66 μg/ml) had little impact of the growth of *P. putida* CA-3 when compared to the untreated control (**Figure 1F**), but no growth was observed over the 36 h period when combined with colistin or polymyxin at 1/4× MIC (0.1 μg/ml and 0.2 μg/ml, respectively). Finally, in the case of *P. aeruginosa* PA-01, polymyxin and colistin at 1/2× MIC in combination with 1/3× MIC nisin was sufficient to completely inhibit growth (**Figures 1G,H**).

### Inhibition of Biofilm Formation with Purified Nisin A and Antibiotic Combinations

**FIGURE 2:**
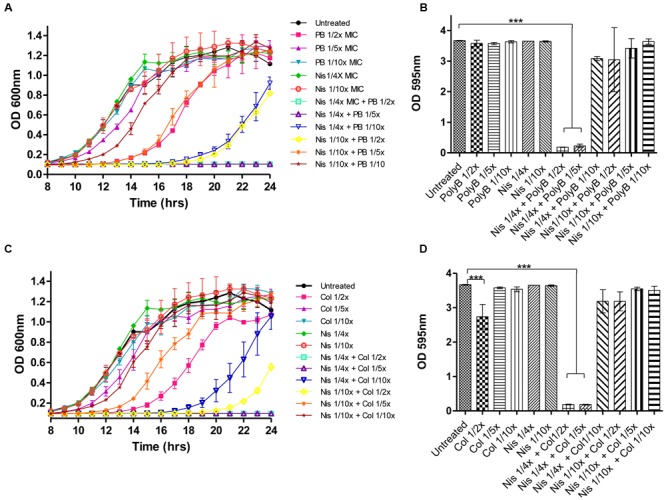
**Growth curve analysis of *P. aeruginosa* PA-01 **(A)** in the presence of nisin (1/4×, 1/10× MIC) and polymyxin B (1/2×, 1/5×, 1/10× MIC) and combinations thereof as carried out in 96 well microtiter plates, followed by crystal violet (CV) staining for the detection of biofilm formation **(B)** and *P. aeruginosa* PA-01 **(C)** in the presence of nisin (1/4×, 1/10× MIC) and colistin (1/2×, 1/5×, 1/10× MIC) and combinations thereof, followed by CV staining (D)**. The means and standard deviations of triplicate determinations are presented. Asterisks indicate statistically significant differences (Student’s t-test) between peptide and antibiotic combinations used at similar concentration (^∗∗∗^p < 0.001).

Prior to carrying out combinatorial experiments against biofilms, the biofilm-forming capabilities of the target strains was assessed and all demonstrated the ability to form biofilms as determined using 96 well flat-bottomed polystyrene plates and staining with crystal violet (data not shown). We employed the same methodology to study the ability of nisin A and colistin or polymyxin combinations to inhibit biofilm formation using *P. aeruginosa* PA-01 as a representative strain. *P. aeruginosa* was selected as a target due to its ability to form biofilms in various environments, its natural resistance to many currently utilized antibiotics and its association with several chronic infectious diseases (Hall-Stoodley et al., 2004). For biofilm prevention studies, colistin, or polymyxin was employed at concentrations 1/2×, 1/5×, or 1/10× MIC (0.78, 0.31, and 0.15 μg/ml, respectively) while nisin was used at 1/4× or 1/10× MIC (50 and 5 μg/ml), as well as combinations thereof. Growth was monitored spectrophotometrically (as Absorbance OD_600_) over 24-h, followed by staining and optical density readings at 595 nm (OD_595_). Notably, none of the antimicrobials inhibited biofilm formation when used alone (**Figures 2B,D**). Indeed, although colistin and polymyxin, when utilized at 1/2× MIC, exerted a significant delay in growth (as evident by the extended lag phase) compared to the untreated control (**Figures 2A,C**), only colistin caused a small reduction in biofilm mass (**Figure 2D**). When lower concentrations (1/10×) of the antibiotics were used, even in combination with nisin, a similar biofilm density was observed to that of the untreated control. However, it was established that combinations of nisin at 1/4× MIC in combination with 1/2× or as little as 1/5× MIC polymyxin B or colistin were able to completely inhibit biofilm formation (^∗∗∗^*p* < 0.001) of *P. aeruginosa* PA-01 due to the inhibition of growth of the bacteria (**Figures 2B,D**). Finally, no significant difference in biofilm density was apparent compared to the untreated control for all other combination of nisin and colistin or polymyxin B.

## Discussion

Infections caused by multi-drug resistant bacteria constitute the leading cause of serious healthcare-associated infections and are responsible for extended periods of hospital stay, severe illness, mortality, and increased economic burden. The polymyxins now play a critical role in the antibiotic arsenal, as they are one of few, and occasionally the sole, antimicrobial agent maintaining efficacy against multi-drug resistant Gram-negative pathogens that frequently cause life threatening infections in the most vulnerable of patient populations. Critically, there are clinical reports confirming that Gram-negative bacteria have developed resistance even to polymyxins (Falagas et al., 2008; Di Pilato et al., 2016), underpinning the necessity for strategies to reduce the effective dose needed for these antibiotics to help prevent or delay the further spread of resistance. The ability of these organisms to form biofilms must also be taken into consideration given the impermeable nature of many biofilms further contributes to resistance. Biofilm suppression can be achieved in three ways, namely: (i) inhibition of the initial planktonic population, (ii) prevention of the initial adhesion of cells to the surface, and (iii) removal of the established biofilm. Because biofilm-associated bacteria are not affected by therapeutically relevant concentrations of antimicrobial agents, anti-biofilm therapies have generally focused on the inhibition of biofilm formation (Dosler and Karaaslan, 2014). Here, we set out to examine, for the first time, the ability of nisin, when used in conjunction with a selection of conventional antibiotics, to control a range of Gram-negative bacteria with the ultimate aim of identifying superior anti-biofilm combinations. Indeed, following MIC determinations and growth curve analysis in the presence of nisin and selected antibiotic combinations, substantial enhanced inhibitory relationships were only observed for nisin in combination with colistin or polymyxin B. The results reveal that sub-inhibitory levels (1/5× MIC and 1/4× MIC for colistin and nisin, respectively) can effectively prevent biofilm formation through total inhibition of growth. Notably, nisin alone had no effect on growth at any of the concentrations utilized. The poor activity of nisin and other lantibiotics toward Gram-negative bacteria is ascribed to the outer membrane (OM) of the Gram-negative cell wall which acts as a physical barrier, impeding the access of the peptides to the cytoplasmic membrane (Nikaido and Vaara, 1985). Indeed, previous studies have confirmed the enhanced efficacy of bioengineered nisin derivatives against Gram-negative bacteria in which the OM no longer functions as an impenetrable barrier following treatment with Polymyxin B nonapeptide (PMBN; Field et al., 2012). The potential benefits associated with identifying antibiotics that function synergistically with nisin are manifold. While antibiotic resistance has become a major obstacle, significant resistance to nisin outside of the laboratory has yet to be reported despite its widespread use as a food preservative (Breukink and de Kruijff, 1999) and thus the use of nisin-antibiotic combinations may prevent/overcome the emergence of resistance. Indeed, such approaches appear particularly promising for combinations of antimicrobials that target different sites. Additionally, combination therapy may permit the dose of the individual antimicrobials to be reduced and consequently counteract the development of drug-resistance in bacteria. Furthermore, the opportunity also exists to combine nisin and colistin/polymyxin with other antibiofilm agents including quorum sensing (QS) inhibitors such as polyphenolic compounds (baicalin hydrate, epigallocatechin) or enzymes for signal molecule destruction that affect biofilms via non-microbicidal mechanisms, but instead target specific molecular pathways that regulate biofilm formation (Brackman and Coenye, 2015). For example, Human HDP LL-37 and the bovine neutrophil peptide indolicidin have previously been shown to prevent *P. aeruginosa* PAO1 biofilm formation at sub-inhibitory concentrations by downregulating the genes essential for cell attachment and biofilm formation (Overhage et al., 2008). Similarly, the antibiotics azithromycin and ceftazidime demonstrated inhibitory effects against *P. aeruginosa* biofilm through the downregulation of a range of QS-regulated virulence factors and adhesion abilities (Skindersoe et al., 2008).

## Conclusion and Perspectives

The incidence of multi-drug resistant bacteria continues unabated despite the best efforts of antimicrobial stewardship and stringent infection control practices in hospitals. In addition to the urgent demand for newer antibiotics, imaginative, and judicious approaches are required to protect the efficacy of the current last resort compounds. The data presented here demonstrates the potential for nisin and conventional antibiotic combinations to act as potent antimicrobial and anti-biofilm agents against Gram-negative pathogens including *P. aeruginosa*. The enhanced activities of combinations of nisin A with both colistin and polymyxin B observed here for the first time to prevent *P. aeruginosa* biofilm formation has significant implications for their future use as novel therapeutics in the treatment of multi-drug resistant bacteria. Furthermore, these data reinforce the idea that bacteriocins can form a novel strategy to prevent adhesion and to control biofilm formation by clinically relevant pathogens and ultimately may facilitate the use of lower concentrations of polymyxin antibiotics in situations where the levels currently exercised are of concern from a toxicity standpoint.

## Author Contributions

DF and NS contributed equally to the manuscript. Conceived and designed the experiments: DF, PC, CH, and RR. Performed the experiments: NS and DF. Analyzed the data: DF, NS, PC, and CH. Contributed reagents/materials/analysis tools: DF, PC, CH, and RR. Wrote the paper: DF, NS, PC, and placecountry-regionCH.

## Conflict of Interest Statement

The authors declare that the research was conducted in the absence of any commercial or financial relationships that could be construed as a potential conflict of interest.

## References

[B1] Bergen P. J. Landersdorfer C. B. Lee H. J. Li J. Nation R. L. (2012). ‘Old’ antibiotics for emerging multidrug-resistant bacteria. *Curr. Opin. Infect. Dis.* 25 626–633. 10.1097/QCO.0b013e328358afe5 23041772 PMC4242550

[B2] Bierbaum G. Sahl H. G. (2009). Lantibiotics: mode of action, biosynthesis and bioengineering. *Curr. Pharm. Biotechnol.* 10 2–18. 10.2174/138920109787048616 19149587

[B3] Boucher H. W. Talbot G. H. Bradley J. S. Edwards J. E. Gilbert D. Rice L. B. (2009). Bad bugs, no drugs: no ESKAPE! An update from the infectious diseases society of America. *Clin. Infect. Dis.* 48 1–12. 10.1086/595011 19035777

[B4] Brackman G. Coenye T. (2015). Quorum sensing inhibitors as anti-biofilm agents. *Curr. Pharm. Des.* 21 5–11. 10.2174/1381612820666140905114627 25189863

[B5] Breukink E. de Kruijff B. (1999). The lantibiotic nisin, a special case or not? *Biochim. Biophys. Acta* 1462 223–234. 10.1016/S0005-2736(99)00208-4 10590310

[B6] Brumfitt W. Salton M. R. Hamilton-Miller J. M. (2002). Nisin, alone and combined with peptidoglycan-modulating antibiotics: activity against methicillin-resistant Staphylococcus aureus and vancomycin-resistant enterococci. *J. Antimicrob. Chemother.* 50 731–734. 10.1093/jac/dkf190 12407132

[B7] Carlet J. Jarlier V. Harbarth S. Voss A. Goossens H. Pittet D. (2012). Ready for a world without antibiotics? The pensières antibiotic resistance call to action. . *Antimicrob. Resist. Infect. Control* 1 1–13. 10.1186/2047-2994-1-11 22958833 PMC3436635

[B8] Cavera V. L. Arthur T. D. Kashtanov D. Chikindas M. L. (2015). Bacteriocins and their position in the next wave of conventional antibiotics. *Int. J. Antimicrob. Agents* 46 494–501. 10.1016/j.ijantimicag.2015.07.011 26341839

[B9] Corvec S. Furustrand Tafin U. Betrisey B. Borens O. Trampuz A. (2013). Activities of fosfomycin, tigecycline, colistin, and gentamicin against extended-spectrum-β-lactamase-producing *Escherichia coli* in a foreign-body infection model. *Antimicrob. Agents Chemother.* 57 1421–1427. 10.1128/aac.01718-12 23295934 PMC3591882

[B10] Cotter P. D. Hill C. Ross R. P. (2005). Bacterial lantibiotics: strategies to improve therapeutic potential. *Curr. Protein Pept. Sci.* 6 61–75. 10.2174/1389203053027584 15638769

[B11] Cotter P. D. Ross R. P. Hill C. (2013). Bacteriocins - a viable alternative to antibiotics? *Nat. Rev. Microbiol.* 11 95–105. 10.1038/nrmicro2937 23268227

[B12] de la Fuente-Núñez C. Hancock R. E. W. (2015). Using anti-biofilm peptides to treat antibiotic-resistant bacterial infections. *Postdoc. J.* 3 1–8. 10.14304/surya.jpr.v3n2.1 PMC4994992 27563687

[B13] Deegan L. H. Cotter P. D. Hill C. Ross P. (2006). Bacteriocins: biological tools for bio-preservation and shelf-life extension. *Int. Dairy J.* 16 1058–1071. 10.1016/j.idairyj.2005.10.026

[B14] Delves-Broughton J. Blackburn P. Evans R. J. Hugenholtz J. (1996). Applications of the bacteriocin, nisin. *Antonie Van Leeuwenhoek* 69 193–202. 10.1007/BF00399424 8775979

[B15] Di Pilato V. Arena F. Tascini C. Cannatelli A. Henrici De Angelis L. Fortunato S. (2016). MCR-1.2: a new MCR variant encoded by a transferable plasmid from a colistin-resistant KPC carbapenemase-producing Klebsiella pneumoniae of sequence type 512. *Antimicrob. Agents Chemother.* 60 5612–5615. 10.1128/aac.01075-16 27401575 PMC4997870

[B16] Dosler S. Gerceker A. A. (2011). In vitro activities of nisin alone or in combination with vancomycin and ciprofloxacin against methicillin-resistant and methicillin-susceptible *Staphylococcus aureus* strains. *Chemotherapy* 57 511–516. 10.1159/000335598 22302084

[B17] Dosler S. Karaaslan E. (2014). Inhibition and destruction of *Pseudomonas aeruginosa* biofilms by antibiotics and antimicrobial peptides. *Peptides* 62 32–37. 10.1016/j.peptides.2014.09.021 25285879

[B18] Falagas M. E. Rafailidis P. I. Matthaiou D. K. Virtzili S. Nikita D. Michalopoulos A. (2008). Pandrug-resistant *Klebsiella pneumoniae, Pseudomonas aeruginosa* and *Acinetobacter baumannii* infections: characteristics and outcome in a series of 28 patients. *Int. J. Antimicrob. Agents* 32 450–454. 10.1016/j.ijantimicag.2008.05.016 18768302

[B19] Field D. Begley M. O’Connor P. M. Daly K. M. Hugenholtz F. Cotter P. D. (2012). Bioengineered nisin a derivatives with enhanced activity against both gram positive and gram negative pathogens. *PLoS ONE* 7:e46884 10.1371/journal.pone.0046884 PMC3466204 23056510

[B20] Field D. Cotter P. D. Hill C. Ross R. P. (2015a). Bioengineering lantibiotics for therapeutic success. *Front. Microbiol.* 6:1363 10.3389/fmicb.2015.01363 PMC4662063 26640466

[B21] Field D. Cotter P. D. Ross R. P. Hill C. (2015b). Bioengineering of the model lantibiotic nisin. *Bioengineered* 6 187–192. 10.1080/21655979.2015.1049781 25970137 PMC4601270

[B22] Field D. Gaudin N. Lyons F. O’Connor P. M. Cotter P. D. Hill C. (2015c). A bioengineered nisin derivative to control biofilms of *Staphylococcus pseudintermedius*. *PLoS ONE* 10:e0119684 10.1371/journal.pone.0119684 PMC4366236 25789988

[B23] Field D. Quigley L. O’Connor P. M. Rea M. C. Daly K. Cotter P. D. (2010). Studies with bioengineered nisin peptides highlight the broad-spectrum potency of nisin V. *Microb. Biotechnol.* 3 473–486. 10.1111/j.1751-7915.2010.00184.x 21255345 PMC3815813

[B24] Gales A. C. Jones R. N. Sader H. S. (2011). Contemporary activity of colistin and polymyxin B against a worldwide collection of Gram-negative pathogens: results from the SENTRY antimicrobial surveillance program (2006–09). *J. Antimicrob. Chemother.* 66 2070–2074. 10.1093/jac/dkr239 21715434

[B25] Gales A. C. Reis A. O. Jones R. N. (2001). Contemporary assessment of antimicrobial susceptibility testing methods for polymyxin B and colistin: review of available interpretative criteria and quality control guidelines. *J. Clin. Microbiol.* 39 183–190. 10.1128/jcm.39.1.183-190.2001 11136768 PMC87699

[B26] Hall-Stoodley L. Costerton J. W. Stoodley P. (2004). Bacterial biofilms: from the natural environment to infectious diseases. *Nat. Rev. Microbiol.* 2 95–108. 10.1038/nrmicro821 15040259

[B27] Healy B. Field D. O’Connor P. M. Hill C. Cotter P. D. Ross R. P. (2013). Intensive mutagenesis of the nisin hinge leads to the rational design of enhanced derivatives. *PLoS ONE* 8:e79563 10.1371/journal.pone.0079563 PMC3823697 24244524

[B28] Kelly D. McAuliffe O. Ross R. P. Coffey A. (2012). Prevention of *Staphylococcus aureus* biofilm formation and reduction in established biofilm density using a combination of phage K and modified derivatives. *Lett. Appl. Microbiol.* 54 286–291. 10.1111/j.1472-765X.2012.03205.x 22251270

[B29] Landman D. Georgescu C. Martin D. A. Quale J. (2008). Polymyxins revisited. *Clin. Microbiol. Rev.* 21 449–465. 10.1128/cmr.00006-08 18625681 PMC2493081

[B30] Lebel G. Piche F. Frenette M. Gottschalk M. Grenier D. (2013). Antimicrobial activity of nisin against the swine pathogen Streptococcus suis and its synergistic interaction with antibiotics. *Peptides* 50 19–23. 10.1016/j.peptides.2013.09.014 24096107

[B31] Liu Y. Y. Wang Y. Walsh T. R. Yi L. X. Zhang R. Spencer J. (2016). Emergence of plasmid-mediated colistin resistance mechanism MCR-1 in animals and human beings in China: a microbiological and molecular biological study. *Lancet Infect. Dis.* 16 161–168. 10.1016/s1473-3099(15)00424-7 26603172

[B32] Marcinkiewicz J. Strus M. Pasich E. (2013). Antibiotic resistance: a “dark side” of biofilm-associated chronic infections. *Pol. Arch. Med. Wewn.* 123 309–313. 23828150

[B33] Naghmouchi K. Baah J. Hober D. Jouy E. Rubrecht C. Sane F. (2013). Synergistic effect between colistin and bacteriocins in controlling Gram-negative pathogens and their potential to reduce antibiotic toxicity in mammalian epithelial cells. *Antimicrob. Agents Chemother.* 57 2719–2725. 10.1128/aac.02328-12 23571533 PMC3716138

[B34] Naghmouchi K. Le Lay C. Baah J. Drider D. (2012). Antibiotic and antimicrobial peptide combinations: synergistic inhibition of *Pseudomonas* fluorescens and antibiotic-resistant variants. *Res. Microbiol.* 163 101–108. 10.1016/j.resmic.2011.11.002 22172555

[B35] Nation R. L. Li J. (2009). Colistin in the 21(st) century. *Curr. Opin. Infect. Dis.* 22 535–543. 10.1097/QCO.0b013e328332e672 19797945 PMC2869076

[B36] Nikaido H. Vaara M. (1985). Molecular basis of bacterial outer membrane permeability. *Microbiol. Rev.* 49 1–32. 2580220 10.1128/mr.49.1.1-32.1985 PMC373015

[B37] Okuda K. Zendo T. Sugimoto S. Iwase T. Tajima A. Yamada S. (2013). Effects of bacteriocins on methicillin-resistant *Staphylococcus aureus* biofilm. *Antimicrob. Agents Chemother.* 57 5572–5579. 10.1128/aac.00888-13 23979748 PMC3811281

[B38] Overhage J. Campisano A. Bains M. Torfs E. C. W. Rehm B. H. A. Hancock R. E. W. (2008). Human host defense peptide LL-37 prevents bacterial biofilm formation. *Infect. Immun.* 76 4176–4182. 10.1128/iai.00318-08 18591225 PMC2519444

[B39] Piper C. Draper L. A. Cotter P. D. Ross R. P. Hill C. (2009). A comparison of the activities of lacticin 3147 and nisin against drug-resistant *Staphylococcus aureus* and *Enterococcus* species. *J. Antimicrob. Chemother.* 64 546–551. 10.1093/jac/dkp221 19561147

[B40] Reffuveille F. de la Fuente-Nunez C. Mansour S. Hancock R. E. (2014). A broad-spectrum antibiofilm peptide enhances antibiotic action against bacterial biofilms. *Antimicrob. Agents Chemother.* 58 5363–5371. 10.1128/aac.03163-14 24982074 PMC4135845

[B41] Rishi P. Preet Singh A. Garg N. Rishi M. (2014). Evaluation of nisin-beta-lactam antibiotics against clinical strains of *Salmonella enterica* serovar Typhi. *J. Antibiot. (Tokyo)* 67 807–811. 10.1038/ja.2014.75 24961707

[B42] Skindersoe M. E. Alhede M. Phipps R. Yang L. Jensen P. O. Rasmussen T. B. (2008). Effects of antibiotics on quorum sensing in *Pseudomonas aeruginosa*. *Antimicrob. Agents Chemother.* 52 3648–3663. 10.1128/aac.01230-07-1237 18644954 PMC2565867

[B43] Tong Z. Zhang Y. Ling J. Ma J. Huang L. Zhang L. (2014). An in vitro study on the effects of nisin on the antibacterial activities of 18 antibiotics against *Enterococcus faecalis*. *PLoS ONE* 9:e89209 10.1371/journal.pone.0089209 PMC3930635 24586598

[B44] Velkov T. Roberts K. D. Nation R. L. Thompson P. E. Li J. (2013). Pharmacology of polymyxins: new insights into an ‘old’ class of antibiotics. *Future Microbiol.* 8 711–724. 10.2217/fmb.13.39 23701329 PMC3852176

[B45] Velkov T. Roberts K. D. Thompson P. E. Li J. (2016). Polymyxins: a new hope in combating Gram-negative superbugs? *Future Med. Chem.* 8 1017–1025. 10.4155/fmc-2016-0091 27328129

[B46] Velkov T. Thompson P. E. Nation R. L. Li J. (2010). Structure—activity relationships of polymyxin antibiotics. *J. Med. Chem.* 53 1898–1916. 10.1021/jm900999h 19874036 PMC2907661

